# ICU-treated influenza A(H1N1) pdm09 infections more severe post pandemic than during 2009 pandemic: a retrospective analysis

**DOI:** 10.1186/s12879-017-2829-3

**Published:** 2017-11-21

**Authors:** Pekka Ylipalosaari, Tero I. Ala-Kokko, Jouko Laurila, Lauri Ahvenjärvi, Hannu Syrjälä

**Affiliations:** 1 Department of Infection Control, Oulu University Hospital, Box 21, FIN-90029 Oulu, OYS Finland; 2 Department of Anesthesiology, Division of Intensive Care, Oulu University Hospital, FIN-90029 Oulu, OYS Finland; 3 Medical Research Center, Research Group of Surgery, Anesthesiology and Intensive Care, University of Oulu, Oulu, Finland; 4 Department of Radiology, Oulu University Hospital, FIN-90029 Oulu, OYS Finland

**Keywords:** Influenza, Critical care, Outcome

## Abstract

**Background:**

We compared in a single mixed intensive care unit (ICU) patients with influenza A(H1N1) pdm09 between pandemic and postpandemic periods.

**Methods:**

Retrospective analysis of prospectively collected data in 2009–2016. Data are expressed as median (25th–75th percentile) or number (percentile).

**Results:**

Seventy-six influenza A(H1N1) pdm09 patients were admitted to the ICU: 16 during the pandemic period and 60 during the postpandemic period. Postpandemic patients were significantly older (60 years vs. 43 years, *p* < 0.001) and less likely to have epilepsy or other neurological diseases compared with pandemic patients (5 [8.3%] vs. 6 [38%], respectively; *p* = 0.009). Postpandemic patients were more likely than pandemic patients to have cardiovascular disease (24 [40%] vs. 1 [6%], respectively; *p* = 0.015), and they had higher scores on APACHE II (17 [13–22] vs. 14 [10–17], *p* = 0.002) and SAPS II (40 [31–51] vs. 31 [25–35], *p* = 0.002) upon admission to the ICU. Postpandemic patients had higher maximal SOFA score (9 [5–12] vs. 5 [4–9], respectively; *p* = 0.03) during their ICU stay. Postpandemic patients had more often septic shock (40 [66.7%] vs. 8 [50.0%], *p =* 0.042), and longer median hospital stays (15.0 vs. 8.0 days, respectively; *p* = 0.006). During 2015–2016, only 18% of the ICU- treated patients had received seasonal influenza vaccination.

**Conclusions:**

Postpandemic ICU-treated A(H1N1) pdm09 influenza patients were older and developed more often septic shock and had longer hospital stays than influenza patients during the 2009 pandemic.

## Background

There are considerable variations in the circulation patterns of the four pandemics that have occurred since 1918, reflecting the unpredictable nature of influenza pandemics. Thus, each new pandemic should be investigated to determine its individual characteristics [1–4]. The most recent one, the 2009 pandemic of H1N1 variant influenza A—influenza A(H1N1) pdm09—had some peculiar features during the first wave, including risk factors for more severe infection, such as obesity and infection during pregnancy (especially during the third trimester) or the postpartum period [4].

Pandemic influenza often appears in waves that differ markedly from one another with respect to variables such as the need for hospitalization or ICU admission [2, 3]. Comparisons of ICU-admitted patients from the 2009 pandemic and the successive waves showed that the patients treated during the second and third waves were older and often had more comorbidities than the patients during the pandemic [2, 5]. According to Martin-Loeches et al., patients infected with influenza A(H1N1) pdm09 during the postpandemic period of 2010–2011 had higher ICU mortality than patients during the 2009 pandemic (30.1 vs. 21.8%, respectively) [5].

Our objective in the present study was to determine whether the clinical features and outcomes of patients with influenza A(H1N1) pdm09 who were treated at our ICU differed depending on whether the patients were infected in the postpandemic period of 2012–2016 or during the 2009–2010 pandemic. We also calculated vaccination rates during the two periods among the influenza patients treated at our ICU and among the general population of the same region.

## Methods

### Study setting and population

The present study was conducted at Oulu University Hospital, a 900-bed tertiary-care teaching hospital. All patients admitted to the ICU for influenza A(H1N1) pdm09 infection during pandemic years 2009–2010 or postpandemic years 2012–2016 were included in the study. We retrospectively analyzed systematically gathered clinical data. The study protocol was approved by the Ethics Committee of Oulu University Hospital. Because the study was epidemiological without any interventions, the requirement for informed consent was waived.

### Study parameters

The following information was collected for all study participants: age; gender; underlying diseases and organ dysfunction on admission as assessed by the Acute Physiology and Chronic Health Evaluation (APACHE II) [6] Sequential Organ Failure Assessment (SOFA) [7], and Simplified Acute Physiology Score **(**SAPS II) [8]. Information regarding the need for vasopressor treatment, mechanical ventilation, renal replacement therapy, or extracorporeal membrane oxygenation (ECMO) was also recorded, as were the highest total, respiratory, and circulatory SOFA scores during ICU treatment. Septic shock was defined as a SOFA circulatory score of 3 or 4. The presence of acuterespiratory distress syndrome (ARDS) was classified according to Berlin ARDS criteria; no, mild, moderate and severe ARDS [9]. The diagnosis of influenza A infection was verified by a real-time polymerase chain reaction (PCR) assay that identifies influenza A, influenza B, and influenza A(H1N1) pdm09 (Xpert Flu®, Cepheid). Lengths of stay in the ICU and at the hospital were recorded, as well as hospital mortality and 6-month mortality. Information regarding influenza vaccination status for both the study participants and for the general population in the Oulu Hospital District region was obtained from the official vaccination registry of the Finnish National Institute for Health and Welfare.

### Data registration and statistical analysis

Values for continuous and ordinal variables are expressed as median (25th–75th percentile) or as number (%). Between-group comparisons were made with Student’s *t* test (continuous variables), Mann-Whitney *U* test (ordinal variables) or Fisher exact test (categorical variables).

We report two-tailed *p* values. The analyses were performed with SPSS software (released 2012; SPSS Statistics for Windows version 21.0, IBM Corporation).

## Results

**Fig. 1:**
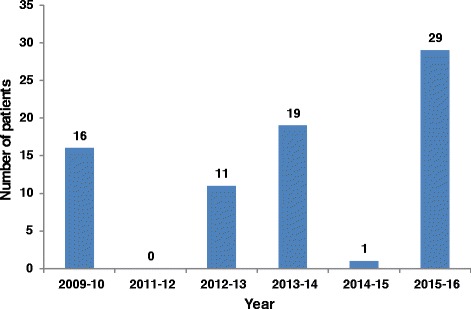
Number of H1N1 influenza patients admitted to ICU during successive influenza seasons

The study population comprised 76 patients with influenza A(H1N1) pdm09 infection treated in our mixed ICU (Table 1). Figure 1 presents the numbers of ICU-treated influenza A(H1N1) pdm09–infected patients in different years. Patients were significantly older during the postpandemic period compared to the pandemic period: 61 (49–66) years vs. 43 (32–53) years, respectively (*p* = 0.001). As Table 1 shows the proportion of patients with epilepsy and other neurological diseases was lower during the postpandemic period than during the pandemic period: 5 (8.3%) vs. 6 (37.5%), respectively (*p* = 0.009). Postpandemic patients had statistically more often cardiovascular diseases: 24 (40%) vs. 1 (6.3%), *p* = 0.015. None of our influenza patients were pregnant. Postpandemic patients had higher scores on APACHE II (17 [13–22] vs. 14 [10–17], *p* = 0.002) and SAPS II (40 [31–51] vs. 31 [25–35], *p* = 0.002) upon admission. Postpandemic patients had a longer median hospital stay: 13.0 (6.9–23.0) days vs. 8.2 (4.6–10) days, respectively (*p* = 0.009). In addition, the 6-month mortality rate was twice as high as for the pandemic patients (25% vs. 12.5%, respectively), although this result was not statistically significant (Table 1).

**Table 1:** Demographic data of pandemic and postpandemic patients

Variables	2009–2010 Pandemic (*n* = 16)	2012–2016 Postpandemic (*n* = 60)	*p* value
Male sex	8 (50)	42 (70)	0.15
Age, years	43 (32–53)	60 (50–65)	< 0.001
BMI	29.7 (24.0–36.1)	27.1 (24.5–32.4)	0.55
BMI 30–40	6 (37)	13 (22)	
BMI > 40	2 (12.5)	6 (10.2)	0.4
Chronic underlying disease	14 (87.5)	49 (83)	0.7
Cardiovascular	1 (6.3)	24 (40)	0.015
COPD/asthma	5 (31,3)	18 (30.0)	>0.9
Diabetes	6 (37.5)	9 (15.0)	0.07
Malignancy or immuno- suppressive medication^a^	1 (6.3)	9 (15.0)	0.7
Epilepsy or other neurological disease	6 (37.5)	5 (8.3)	0.009
APACHE II score on admission	14 (10–17)	17 (13–22)	0.002
SAPS II score on admission	31 (25–35)	40 (31–51)	0.002
SOFA score on admission	5 (3–7)	6 (3–9)	0.31
SOFA score max	5 (4–9)	9 (5–12)	0.03
TISS score	34 (28–42)	39 (28–53)	0.40
LOS in ICU, days	1.9 (1.1–4.8)	4.3 (2.3–10.7)	0.31
LOS in hospital, days	8.2 (4.6–10)	13.0 (6.9–23.3)	0.009
ICU mortality	0	6 (10)	0.33
Hospital mortality	0	10 (16.7)	0.11
6-month mortality	2 (12.5)	15 (25)	0.5

*APACHE* Acute Physiology and Chronic Health Evaluation, *BMI* body mass index, *ICU* intensive care unit, *LOS* length of stay, *SAPS* Simplified Acute Physiology Score, *SOFA* Sequential Organ Failure Assessment, *TISS* Therapeutic Intervention Scoring System

^a^lymphoma, *n =* 2; leukemia, *n =* 2; carcinoma, *n =* 1; long-lasting immunosuppressive medication, *n =* 5

Values are presented as median (25th–75th percentile) or number (percentage) of patients. Chronic underlying diseases included previously diagnosed chronic obstructive pulmonary disease, ischemic heart disease, chronic hepatic disease, chronic renal disease, previous stroke or transient ischemic attack, diabetes, and malignancy or immunosuppressive medication. Some patients had more than one chronic underlying disease

Postpandemic influenza patients were more likely to have dyspnea: 86.7% vs. 62.5%, respectively (*p* = 0.037) (Table 2). During both periods, most patients had pulmonary opacities, which were typically bilateral (Table 2). During the pandemic 11 of the patients (68.7%) had ARDS, while the corresponding figure during postpandemic period was 43 (71.7%), *p* = 0.69. Bacteria were cultured from bronchoalveolar lavation or tracheal aspiration for every patient who was admitted to the ICU with pneumonia. Mixed influenza and bacterial respiratory infections occurred more often among pandemic patients than among postpandemic patients: 7 (43.8%) vs. 9 (15%), respectively (*p* = 0.033).

**Table 2:** Clinical data of pandemic and postpandemic patients

Variables	2009–2010 Pandemic (*n* = 16)	2012–2016 Postpandemic (*n* = 60)	*p* value
Delay from symptoms to hospital admission, hours	108.0 (48–168)	72 (24–168)	0.37
Delay from hospital admission to ICU admission, days	1.9 (1.12–4.78)	1.04 (0.07–2.18)	0.25
Principal symptoms			
fever	10 (66.7)	43(71.7)	0.06
dyspnea	10 (62.5)	52 (86.7)	0.037
cough	11 (68.8)	28 (46.7)	0.16
gastrointestinal	5 (31.3)	13 (21.7)	0.51
neurological	1 (6.3)	15 (25)	0.17
PaO_2_/FiO_2_, kPa	33.4 (16.5–40.6)	26.3 (15.0–42.6)	0.41
Pneumonia on chest x-ray	11 (68.8)	53 (88.3)	0.11
Bilateral opacities	11 (100.0)	49 (92.5)	0.16
ARDS			0.69
No	5 (31.3)	17 (28.3)	
Mild	2 (12.5)	3 (5.0)	
Moderate	3 (18.8)	14 (23.3)	
Severe	6 (37.5)	26 (43.3)	
Presence of bacteremia on admission	0	5 (10)	0.33
Mixed bacterial infection	7 (43.8)	9 (15)	0.033
Antiviral treatment on ICU admission	13 (81.3)	45 (75)	0.75
Bacterial antibiotics	15 (93.8)	48 (82)	0.48

*ARDS* acute respiratory distress syndrome, *ICU* intensive care unit

PaO2/FiO2 ratio is the ratio of arterial oxygen partial pressure to fractional inspired oxygen

Values are presented as median (25th–75th percentile) or as number (percentage) of patients

Postpandemic patients also had significantly higher concentrations of lactate upon ICU admission: 1.38 mmol/l vs. 1.02 mmol/l, respectively (*p* = 0.04) (Table 3). Postpandemic patients had a higher maximum SOFA score during their ICU stay: 9 (5–12) vs. 5 (4–9), *p* = 0.03. Influenza A(H1N1) pdm09 patients treated in the postpandemic period required vasopressor treatment more than twice as often as those treated during the pandemic period: 63.3% vs. 31.0% (*p* = 0.03), respectively (Table 4). Postpandemic patients also had septic shock significantly more often, according to SOFA cardiovascular scores (*p* = 0.042). The severity of respiratory organ dysfunction did not differ significantly between the two time periods. During postpandemic period four patients with severe ARDS needed ECMO treatment, because conservative treatment of hypoxia was unsuccessful (Table 4).

**Table 3:** Main laboratory values of pandemic and postpandemic patients upon admission to ICU

Variables	2009–2010 Pandemic (*n* = 16)	2012–2016 Postpandemic (*n* = 60)	*p* value
WBC, 10^9^/l	8.6 (6.2–12.5)	6.9 (4.1–9.6)	0.48
Platelets, 10^9^/l	192 (151–278)	167 (107.5–220)	0.14
C-reactive protein, mg/l	109 (29–145)	104 (51–200)	0.22
Lactate, mmol/l	1.02 (0.88–1.46)	1.38 (1.05–2.18)	0.04
Creatinine, μmol/l	64.5 (47.5–73)	77 (59–120)	0.75

*WBC* white blood cells

Values are presented as median (25th–75th percentile)

**Table 4:** Specific treatment of pandemic and postpandemic patients during their stay in ICU

Variables	2009–2010 Pandemic (*n* = 16)	2012–2016 Postpandemic (*n* = 60)	*p* value
Invasive ventilation	8 (50)	37 (61.7)	0.40
Noninvasive ventilation	9 (56)	42 (70)	0.37
Vasopressor treatment	5 (31)	38 (63.3)	0.03
Renal replacement therapy	1 (6.3)	11 (18.3)	0.44
Duration of invasive ventilation, hours	36.7 (17.5–176.5)	114 (24–193.5)	0.43
Duration of noninvasive ventilation, hours	16 (15–42)	27.7 (7.8–53.6)	0.45
ECMO	0	4 (6.7)	0.57
SOFA circulation			0.042
0 points	3 (18.8)	3 (5.0)	
1 point	8 (50.0)	19 (31.7)	
2 points	0	0	
3 points	1 (6.3)	16 (26.7)	
4 points	4 (25.0)	22 (36.7)	
SOFA respiratory			0.38
0 points	1 (6.3)	1 (1.7)	
1 point	1 (6.3)	7 (11.7)	
2 points	6 (37.5)	12 (20.0)	
3 points	3 (18.8)	16 (26.7)	
4 points	5 (31.3)	24 (40.0)	

*ECMO* extracorporeal membrane oxygenation, *ICU* intensive care unit, *SOFA* Sequential Organ Failure Assessment

Values are presented as median (25th–75th percentile) or as number (percentage) of patients

**Fig. 2:**
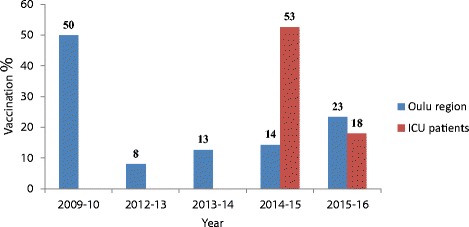
Vaccination rates in general population of the Oulu Hospital District region compared to vaccination rates of ICU-treated patients during successive influenza seasons

During the influenza period of 2013–2014, ten out of 18 patients (52.6%) were vaccinated (Fig. 2). For 2015–2016, vaccination data were available for 28 out of 29 patients (96.6%); only five (17.9%) of them were vaccinated (Fig. 2). Two of these vaccinated patients were older than 65 years. The vaccination rate in the general population of the Oulu University Hospital District region was higher during the pandemic period than thereafter (Fig. 2).

## Discussion

Our study revealed that during the postpandemic period, patients with influenza A(H1N1) pdm09 infection admitted to the our ICU were older and had higher APACHE II and SAPS scores. They also experienced septic shock more frequently and had longer hospital stays. Furthermore, they had poor compliance with seasonal influenza vaccination.

In our series, patients admitted to the ICU during the postpandemic period were more severely ill upon admission and developed septic shock more frequently. Increased severity of disease during the postpandemic second and third waves has been noted previously [1, 2]. In Finland, the influenza A(H1N1) pdm09 viruses circulating in 2013 and 2014 were found to have antigenically drifted from the 2009 isolate, with several mutations. According to whole-genome sequencing, the 2013–2014 viruses were approximately 1.3% different at the amino acid level from the 2009 isolate [10]. These changes may at least partially explain the more virulent postpandemic disease. Furthermore, in Great Britain, during the second and third waves of the pandemic/postpandemic of influenza A(H1N1) pdm09, the virus replicated more rapidly in human airway epithelial cells than during the first wave of the pandemic [11]. The more rapid replication in human cells may have facilitated viral replication, leading to the more severe influenza cases seen during the postpandemic period.

During the last epidemic of 2015–2016, less than one-fifth of the patients treated at our ICU were vaccinated. Moreover, two of the five vaccinated patients were older than 65 years. At that age, standard-dose influenza vaccination is not as effective as high-dose vaccination [12–14], which is unavailable in Europe. Not being vaccinated is an independent risk factor for severe influenza in hospitalized patients [15]. Influenza vaccination decreases the risk of severe consequences (i.e., the need for ICU treatment and death in hospital or within 30 days after admission), at least in hospitalized patients >65 years old [16, 17]. On the other hand, in a recent study, influenza vaccination did not decrease mortality among severely ill ICU patients. This was surprising because the vaccine strain was identical with the H1N1 virus circulating in the community [18]. In another study, only influenza patients aged 50–64 years had a shorter ICU stay if they had been vaccinated [19]. In our ICU, the patients with influenza had underlying diseases and poor compliance with influenza vaccination. More systematic studies are required to evaluate whether influenza vaccination effectively decreases the need for ICU treatment of influenza patients.

Our influenza patients were severely ill. Postpandemic patients had more often ARDS than did pandemic patients (83.3 vs. 68.7%). These figures are clearly higher than reported, for example, in a Spanish study, where 9% of the pandemic patients and 26% of the postpandemic patients had ARDS, or from the United States during pandemic, where 38% of ICU-treated influenza A(H1N1) pdm09 patients developed ARDS [2, 20]. One reason for the difference in the incidences of ARDS between our and earlier results may be due to different ARDS criteria; earlier studies did not specify the criteria, and we used Berlin ARDS criteria [9].

However, in our ICU series, the ICU mortality of patients infected with influenza A(H1N1) pdm09 was low; 0% during the pandemic and 10% during the postpandemic period. These figures are lower than those observed in a multicenter Spanish study (30.1 vs. 21.8%, respectively) [2] and in a single-center Turkish study (26% among the pandemic patients) [21]. A report of a US case series showed that of the 108 adult patients admitted to an ICU during pandemic 28 (26%) died [20].

For more than 75% of patients during both periods antiviral treatment was started in the emergency room or in the ward before ICU admission; for the remaining patients their antiviral treatment was started on ICU admission. During seasonal influenza epidemics, we initiate antiviral treatment for all of our ICU patients with respiratory symptoms. During seasonal influenza epidemics, we initiate antiviral treatment for all of our ICU patients with respiratory symptoms. Antiviral treatment is halted, if the PCR for influenza yields a negative result.

The benefits of antiviral treatment are controversial in the literature. A recent systematic analysis of influenza patients found only small benefits for either oseltamavir or zanamavir in the prophylaxis or treatment of influenza [22]. However, they did not perform a subgroup analysis of neuraminidase inhibitor treatment of critically ill patients [22]. Muthuri et al. reported that early versus later neuraminidase inhibitor treatment reduced the likelihood of mortality and the need for ventilator support in influenza patients with documented pneumonia [23]. Our results, which suggest an association between early initiation of antiviral treatment and good outcome, support the active use of antiviral treatment for ICU populations with PCR-verified influenza during influenza epidemics.

In concordance with the literature, our postpandemic patients infected with influenza A(H1N1) pdm09 were older [1, 2]. Moreover, most of them had chronic underlying diseases (see Table 1). There were significantly more neurological diseases among pandemic patients, while postpandemic patients had more cardiovascular diseases, which may have been a reflection of the average age in both groups. Postpandemic patients were more severely ill upon admission in terms of risk stratification score, which has been noted in other studies [1, 2, 6]. It has been earlier shown that the presence of bacterial co-infection has been similar during the pandemic and postpandemic period [2]. In our series, pandemic patients had almost three times more often (2.9) mixed influenza and bacterial co-infections than postpandemic patients (*p* = 0.033).

The strengths of the present study include the 6-year postpandemic follow-up period, which enabled us to investigate long-term postpandemic phenomena. Another strength of our study is that the ICU admission criteria did not differ greatly during the study period. Moreover, we had the same multidisciplinary ICU team of intensivists, infectious disease physicians, a cardiologist, and a chest radiologist during the 6-year period. However, our series is a relatively small single-center study; thus, our results may not be directly generalizable to other ICUs that treat patients with influenza A(H1N1) pdm09.

## Conclusions

During 2012–2016, patients admitted to the ICU with influenza A(H1N1) pdm09 had more severe disease and required vasopressor treatment for septic shock twice as often as those admitted to the ICU during the 2009 influenza A(H1N1) pdm09 pandemic. Moreover, the vaccination rate was lower among these postpandemic patients.

## Abbreviations

APACHE: Acute Physiology and Chronic Health Evaluation
ARDS: Acute respiratory distress syndrome
BMI: Body mass index
ECMO: Extracorporeal membrane oxygenation
ICU: Intensive care unit
LOS: Length of stay
PCR: Polymerase chain reaction
SAPS II: Simplified Acute Physiology Score
SOFA: Sequential Organ Failure Assessment
TISS: Therapeutic Intervention Scoring System
WBC: White blood cell
